# Significant association between osteoporosis and hearing loss: a systematic review and meta-analysis^☆^

**DOI:** 10.1016/j.bjorl.2016.08.012

**Published:** 2016-09-12

**Authors:** Sikarin Upala, Pattara Rattanawong, Wasawat Vutthikraivit, Anawin Sanguankeo

**Affiliations:** a Bassett Medical Center and Columbia University College of Physicians and Surgeons, Department of Internal Medicine, Cooperstown, United States; b Mahidol University, Faculty of Medicine Siriraj Hospital, Department of Preventive and Social Medicine, Bangkok, Thailand; c University of Hawaii, Department of Internal Medicine, Honolulu, United States

**Keywords:** Osteoporosis, Hearing loss, Meta-analysis, Osteoporose, Perda auditiva, Metanálise

## Abstract

**Introduction:**

There is inconclusive evidence whether osteoporosis increases risk of hearing loss in current literature.

**Objective:**

We conducted this meta-analysis to determine whether there is an association between hearing loss and osteoporosis.

**Methods:**

This systematic review and meta-analysis was conducted from studies of MEDLINE, EMBASE, and LILACS. Osteoporosis was defined as having a bone mineral density with a *T*-score of less than −2.5 standard deviation. The outcome was hearing loss as assessed by audiometry or self-reported assessment. Random-effects model and pooled hazard ratio, risk ratio, or odds ratio of hearing loss with 95% confidence intervals were compared between normal bone mineral density and low bone mineral density or osteoporosis.

**Results:**

A total of 16 articles underwent full-length review. Overall, there was a statistically significant increased odds of hearing loss in the low bone mineral density or osteoporosis group with odds ratio of 1.20 (95% confidence intervals 1.01–1.42, *p* = 0.04, *I*^2^ = 82%, P_heterogeneity_ = 0.01). However, the study from Helzner et al. reported significantly increase odds of hearing loss in the low bone mineral density in particular area and population included femoral neck of black men 1.37 (95% confidence intervals 1.07–1.76, *p* = 0.01) and total hip of black men 1.36 (95% confidence intervals 1.05–1.76, *p* = 0.02).

**Conclusion:**

Our study proposed the first meta-analysis that demonstrated a probable association between hearing loss and bone mineral density. Osteoporosis could be a risk factor in hearing loss and might play an important role in age-related hearing loss.

## Resumo

**Introdução:**

Há evidências inconclusivas sobre se a osteoporose aumenta o risco de perda auditiva na literatura atual.

**Objetivo:**

Realizamos esta metanálise para determinar se existe uma associação entre perda auditiva e osteoporose.

**Método:**

Revisão sistemática e metanálise foram realizadas a partir de estudos do Medline, Embase e Lilacs. A osteoporose foi definida como tendo uma densidade mineral óssea com um escore T de menos que -2,5 DP. O desfecho foi a perda auditiva, avaliada por audiometria ou avaliação autorrelatada. O modelo de efeitos aleatórios e risco combinado, razão de risco e odds ratio de perda auditiva com Intervalos de Confiança de 95% foram comparados entre densidade mineral óssea normal e densidade mineral óssea baixa ou osteoporose.

**Resultados:**

No total, 16 artigos foram submetidos a revisão completa. Em geral, houve aumento estatisticamente significativo da probabilidade de perda auditiva no grupo de baixa densidade mineral óssea ou no grupo de osteoporose com odds ratio de 1,20 (intervalo de confiança de 95% 1,01-1,42, *p* = 0,04, *p* = 82%, P_heterogeneidade_ = 0,01). No entanto, o estudo de Helzner et al. relatou aumento significativo da probabilidade de perda auditiva na densidade mineral óssea baixa, em determinada área e população que incluiu colo femoral de homens negros 1,37 (intervalo de confiança de 95% 1,07-1,76, *p* = 0,01) e quadril total de homens negros 1,36 (intervalo de confiança de 95% 1,05-1,76, *p* = 0,02).

**Conclusão:**

Nosso estudo propôs a primeira metanálise que demonstrou uma provável associação entre perda auditiva e densidade mineral óssea. A osteoporose pode ser um fator de risco para perda auditiva e pode desempenhar um papel importante na perda de audição relacionada com a idade.

## Introduction

Hearing loss is a common chronic condition of a disability estimated at 24.9 million people worldwide. It was reported by The World Health Organization as one of the leading causes of years lived with disability.^1^ The estimated prevalence of hearing loss was 30% in the population over 65 years old and 50% in the population over 75 years old.^2^, ^3^ Moreover, hearing loss is also associated with decreasing quality of life and functional outcomes including social isolation, depression, safety issues, mobility limitations, reduced income and employment opportunities.^4^, ^5^, ^6^, ^7^ Risk factors influence to the degree and rate of deterioration of hearing loss include aging, genetic susceptibility, ototoxic medication exposure, otological disorders, smoking, and occupational and leisure noise exposure.^6^, ^8^, ^9^, ^10^

Osteoporosis has also been identified in some studies as a risk factor of hearing loss. The underlying mechanism of hearing loss in osteoporosis is complex and undetermined. Some studies purposed that a possible underlying mechanism is systemic demineralization of the skeletal system in osteoporosis includes temporal bone, which contains the cochlea capsule and the conductive system.^11^, ^12^, ^13^ However, there were controversies and inconsistent results from other studies that showed non-significant association between osteoporosis and hearing loss. The accuracy of the results was limited due to the sample sizes of the study populations.^2^ Therefore, we conducted this meta-analysis to determine whether there is an association between hearing loss and low bone mass or osteoporosis.

## Materials and methods

This systematic review and meta-analysis was conducted and reported according to the Meta-analysis Of Observational Studies in Epidemiology statement^14^ and was registered in PROSPERO (registration number: CRD42015024987).

### Search strategy

Two authors (AS, SU) independently searched published studies indexed in the MEDLINE, EMBASE, and LILACS (Literatura Latino Americana em Ciências da Saúde) from their date of inception to November 2015. References of all selected studies were also examined. The following main search terms were used: osteoporosis, osteopenia, bone density, bone mass, bone loss, hearing loss, audiometry, otoacoustic. The full search strategy was detailed in Appendix 1.

### Inclusion and exclusion criteria

Articles were considered eligible for inclusion if the following criteria were met: (1) published observational studies including cross-sectional, cohort, and case–control studies; (2) study in adults age 18 years or older; (3) clear methods of assessment of bone mineral density and hearing status were described; (4) clear diagnostic criteria for osteoporosis and hearing loss were reported; and (5) association of low BMD or osteoporosis and hearing loss was reported as either adjusted or unadjusted hazard ratios (HRs), relative risks (RRs), or odds ratios (ORs) with associated 95% confidence intervals (CIs), or hearing sensitivity in decibels. Exclusion criteria were (1) reviews, case reports, abstracts, and unpublished studies, (2) studies without specific sample origins, (3) data in the study was not presented clearly enough, and (4) participants with known otosclerosis.

Osteoporosis was defined as having a bone mineral density (BMD) with a *T*-score of less than −2.5 SD as measured by dual-energy X-ray absorptiometry or other standard technique at anatomical bone sites including lumbar spine, femoral neck, and total hip. The main outcome of this study was hearing loss as assessed by audiometry or self-reported assessment. We used the definition of hearing loss (conductive, sensorineural, or mixed) as described by each study.

### Data extraction

Two authors (AS and SU) independently reviewed titles and abstracts of all citations that were identified. After all abstracts were reviewed, data comparisons between the two investigators were conducted to ensure completeness and reliability. The inclusion criteria were independently applied to all identified studies. Differing decisions were resolved by consensus.

Full-text versions of potentially relevant papers identified in the initial screening were retrieved. If multiple articles from the same study were found, only the article with the most complete data was included. Data concerning study design, participant characteristics, source of data, comorbidities, methods of assessing BMD and hearing impairment, outcome assessment, and factors adjusted in multivariable analysis were independently extracted.

### Assessment of quality

A subjective assessment of methodological quality for observational studies was evaluated by two authors (AS and SU) using the Newcastle–Ottawa Scale (NOS). The NOS is a quality assessment tool for non-randomized studies. The NOS includes eight items, categorized into three dimensions of selection, comparability, and outcome. For each dimension, a list of response options is provided. Scoring is based on a semi-quantitative assessment of study quality. The highest quality studies are scored a maximum of one point for each item. However, there is an exception of the item related to comparability that allows the assignment of two points. The range of NOS is between zero up to nine points.^15^ A total score of 3 or less was considered poor, 4–6 was considered moderate, and 7–9 was deemed high quality. We excluded studies from our meta-analysis if they had poor quality. Discrepant opinions between authors were resolved by consensus.

### Statistical analysis

We performed meta-analysis of the included studies using Comprehensive Meta-Analysis 3.3 software from Biostat, Inc. We used a random-effects model if there was high heterogeneity (*I*^2^ > 50%) and fixed-effects model if there was low heterogeneity (*I*^2^ < 50%). We calculated pooled HR, RR, or OR of hearing loss with 95% confidence intervals (CI) comparing between participants with normal BMD and with low BMD or osteoporosis at each anatomical site and with any anatomical sites. We also calculated pooled mean difference (MD) with 95% CI of hearing sensitivity in each frequency comparing between the normal BMD group and the low BMD group. We excluded studies from meta-analysis and only presented the result with narrative description when there were not sufficient comparable data available for outcome of interest. The heterogeneity of effect size estimates across these studies was quantified using the *Q* statistic, its *p*-value, and *I*^2^ (*p* < 0.10 was considered significant). Subgroup analysis by site of BMD was performed to find the source of heterogeneity. Publication bias was assessed using funnel plot and Egger's regression test.

## Results

### Description of included studies

**Figure 1:**
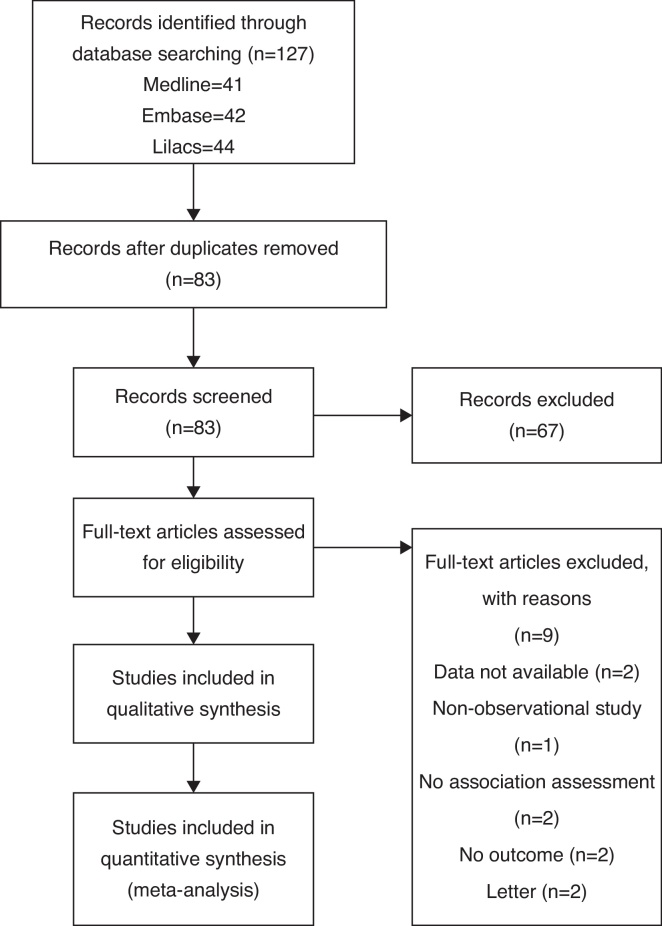
Results of information search.

The initial search yielded 83 articles (Fig. 1); 67 articles were excluded because they were not original observational studies (23 articles), did not have BMD data (12 articles), did not have hearing loss data (6 articles), or did not measure association between BMD and hearing loss (26 articles). A total of 16 articles underwent full-length review. Data were extracted from eight studies involving 52,828 participants who had bone mineral density and hearing status assessed.^2^, ^12^, ^13^, ^16^, ^17^, ^18^, ^19^, ^20^

Most of them had cross-sectional design; others were prospective cohort, retrospective cohort, and case–control studies. Included studies were from Turkey, USA, and Korea. These included national population-based studies from two nations. All participants were assessed by bone mineral density by standardized methods (dual energy X-ray absorptiometry or DXA). Sites of BMD measurement were femur, lumbar spine, head, and methods of assessing hearing status included audiometry, otoscopic examination, and self-reported. The characteristics of the eight extracted studies included in this review are outlined in Table 1.

**Table 1:** Characteristics of included studies.

Study, year,	Country	Design	Characteristics		Participants (*n*)	Outcome definition	Factors adjusted in multivariate model
			Age	Female (%)			
Clark K. et al., 1995	USA	Cross-sectional descriptive study	Women aged 60–85 years	100	369	40 dB HL at 1000 and 2000 Hz in one ear. 40 dB HL at 1000 or 2000 Hz in both ears.	Age and community of residence
Helzner EL et al., 2004	USA	Cross-sectional study	Women aged 65 years or older	100	6474	Mild = hearing at the more intense level (40 dB HL), but not the less intense level (25 dB HL). Significant = failing to hear at both intensity levels.	Age, BMI, estrogen use, sedative use, antidepressant use
Kim SH et al., 2002	South Korea	Cross-sectional	Women aged 50 years or older	100	1830	40 dB HL at 1000 and 2000 Hz in one ear. 40 dB HL at 1000 or 2000 Hz in both ears.	Age, bone mineral density, and serum concentration of estradiol
Kahveci OK et al., 2014	Turkey	Case–control study	Osteoporosis, osteopenia patients and controls was 26–85, 22–83 and 50–68 years, respectively	100	125	Sensorineural hearing loss = bone conduction > 25 dB HL without air-bone gap. Conductive hearing loss = normal bone conduction threshold average, but an air-bone gap > 10 dB HL.	
Mendy A et al., 2014	USA	Cross-sectional survey of the civilian, noninstitutionalized U.S. population	Aged 40 years and older			No hearing trouble. Little hearing trouble. Significant hearing trouble.	Age, gender, race/ethnicity, education level, body mass index
Helzner EP et al., 2005	USA	Prospective cohort study	Aged 70–79	47.27	2052	Hearing loss = pure tone average (PTA) > 25 dB HL in the worse ear. Conductive hearing loss = 15 dB or greater or greater air-bone gap at any two consecutive frequency tested (0.5, 1, 2 and 4 kHz) in the worse ear.	Age, history of ear surgery, alcohol use, diabetes, smoking, cardiovascular disease, cerebrovascular disease, mini-mental score, hypertension, occupational noise exposure, use of salicylates
Yeh MC et al., 2015	Taiwan	Retrospective cohort study	All Age	89.79	42,640	SSNHL = failing to hear at least one frequency at both intensity levels.	Age group, sex, diabetes, hypertension, CAD, chronic kidney disease, income, and area.
Ozkiris M et al., 2013	Turkey	Cross-sectional	Age range from 50 to 55 years	100	75	Mean values of air and bone conduction at each frequency No definition of SSNHL.	No adjust

CAD, coronary artery disease; dB HL, decibel hearing level; SSNHL, sensorineural hearing loss.

### Quality assessment of included studies

The quality of nine cross-sectional, three cohort and, two case–control studies were evaluated by NOS (Table 1). Total score ranged from 3 to 8. Two studies had low quality (total score = 3) and were exclude from the meta-analysis.

### Meta-analysis results

**Figure 2:**
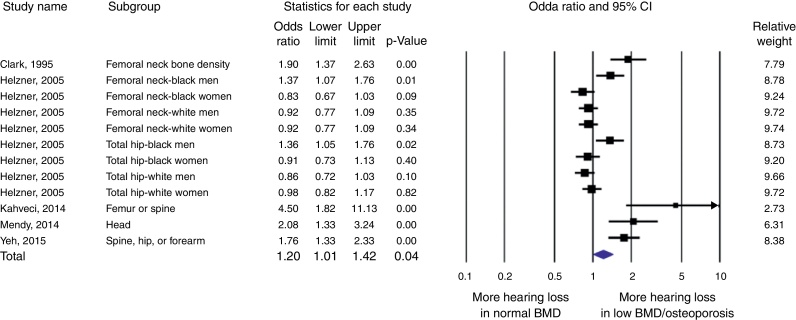
Forest plot of studies comparing odds of hearing loss in patients who had low bone mineral density or osteoporosis and control. A diamond data marker represents the overall odds ratios and its 95% CI.

Five studies (2, 12, 13, 16, 20) were included in the meta-analysis of hearing loss. There was a statistically significant increased odds of hearing loss in the low BMD or osteoporosis group with OR of 1.20 (95% CI 1.01–1.42, *p* = 0.04, *I*^2^ = 82%, P_heterogeneity_ = 0.01) (Fig. 2). The study from Clark et al., Kahveci et al., Mendy et al., and Yeh et al. all reported significantly increased odds of hearing loss in the low BMD group with OR of 1.90 (95% CI 1.37–2.63, *p* < 0.01), 4.50 (95% CI 1.82–11.13, *p* < 0.01), 2.08 (95% CI 1.33–3.24, *p* < 0.01), and 1.76 (95% CI 1.33–2.33, *p* < 0.01), respectively. However, the study from Helzner et al. reported significantly increased odds of hearing loss in the low BMD group, in particular the area and population included the femoral neck of black men 1.37 (95% CI 1.07–1.76, *p* = 0.01) and total hip of black men 1.36 (95% CI 1.05–1.76, *p* = 0.02).

### Sensitivity analysis

To assess the stability of the results of the meta-analysis, we conducted a sensitivity analysis by excluding one study at a time. None of the results was significantly altered, indicating that our results were robust.

### Publication bias

**Figure 3:**
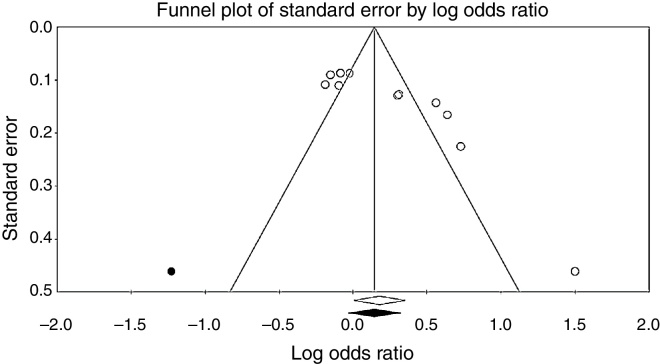
Funnel plot assessing publication bias.

To investigate potential publication bias, we examined the contour-enhanced funnel plot of the included studies in assessing change in log OR of hearing loss (Fig. 3). The vertical axis represents study size (standard error) while the horizontal axis represents effect size (log odds ratio). From this plot, bias is not present because there is symmetrical distribution of studies on both sides of the mean. The Egger's test was non-significant (*p* = 0.36). Using the trim and fill methods in the random-effects model, there was no difference of the imputed OR (1.38) and its 95% CI (1.08–1.7).

## Discussion

Since the association between bone mass and hearing loss from previous studies are inconsistent, we conducted the first meta-analysis of the association between bone mineral density and hearing loss. According to our meta-analysis of 5 studies from different countries, age groups, genders and races, we found that a decrease in BMD or osteoporosis was significantly associated with hearing loss.

Age related hearing loss or “presbycusis” is caused by multifactorial etiologies. A recent study purposed that demineralized petrous temporal bone in addition to age-related bone mass loss could be the cause of developing presbycusis.^11^, ^21^ Interestingly, in Paget disease of the bone, demineralization of the cochlear bone is associated with hearing loss. However, the etiology of the association is unclear.^22^ In concordance with demineralization in Paget disease of the bone, a study conducted in otoslcerosis patients by high-resolution computed tomographic evaluation of the cochlear capsule showed decreased BMD at specific locations on the cochlear capsule. Therefore, decreasing BMD physiologically associates with hearing loss.

The etiology of Paget disease of the bone and otosclerosis share similar pathogenesis in the lateral wall of the cochlea, where the abnormal bone remodeling manipulates the change in ion and fluid hemostasis in perilymphatic space of the cochlea.^23^ However, there are several unique characteristics of the pathologic change in otosclerosis, including fibrous thickening and loss of cochlear blood vessels, spiral ligament hyalinization and stria vascularis atrophy.^24^

Therefore, imbalance in bone formation and bone resorption from osteoporosis may play an important role in dysfunctional ionic metabolism leading to sensory neural hearing loss.

Normally, BMDs at peripheral sites has a strong correlation with measurements at hip and spine. The correlation coefficients between peripheral sites and central sites is between 0.6 and 0.70 (25). However, some populations whose peripheral measurements are normal could have osteoporotic hip or spine; for example, the postmenopausal woman with significant osteoporotic risk factors.^25^ Therefore, different sites of BMD measurement from each study may not accurately reflect total body BMD. With limited results from previous studies, our study demonstrated the first meta-analysis of correlation between hearing loss and BMD. Every study that was included in our meta-analysis did not report total body BMD. Nevertheless, our meta-analysis has raised the concern of hearing loss in osteoporosis, since our result is the strongest evidence of the association between hearing loss and osteoporosis ever reported. Therefore, to evaluate more evidence of the association, further cohort studies of the association between total body BMD and hearing loss should be evaluated.

The limitations of our study include different hearing loss outcomes and different sites of BMD measurement from different studies. Hearing loss outcomes were determined in different aspects of measurement including audiometry and patient self-evaluation. Variation in the outcome of hearing loss could potentially alter the results and conclusion. Since different sites of BMD measurement may not be accurate as total BMD, the interpretation of our study may be limited.

## Conclusion

In conclusion, our study proposed the first meta-analysis that demonstrated a probable association between hearing loss and BMD. Osteoporosis could be a risk factor in hearing loss and might play an important role in age-related hearing loss.

## Ethical approval

This article does not contain any studies with human participants or animals performed by any of the authors.

## Conflicts of interest

The authors declare no conflicts of interest.

## Figures and Tables

**Table:** 

**MEDLINE**
1) Hearing loss.mp. or exp Hearing Loss/
2) Audiometry.mp. or exp Audiometry/
3) Otoacoustic.mp. [mp = title, abstract, original title, name of substance word, subject heading word, keyword heading word, protocol supplementary concept word, rare disease supplementary concept word, unique identifier].
4) Exp Osteoporosis/or osteoporosis.mp.
5) Osteopenia.mp.
6) Exp Bone Density/or bone density.mp.
7) Bone mass.mp.
8) Bone loss.mp.
9) BMD.mp.
10) Bone mineral density.mp.
11) 1 or 2 or 3
12) 4 or 5 or 6 or 7 or 8 or 9 or 10
13) 11 and 12
14) limit 13 to humans
**EMBASE**
((’osteoporosis’/exp or ‘osteoporosis’ and [embase]/lim) or (osteopenia and [embase]/lim) or (’bone density’ and [embase]/lim) or (’bone mass’ and [embase]/lim) or (’bone loss’ and [embase]/lim) or (bmd and [embase]/lim)) and ((’hearing loss’/exp or ‘hearing loss’ and [embase]/lim) or (’audiometry’/exp or ‘audiometry’ and [embase]/lim)) and [humans]/lim) and [embase]/lim not [medline]/lim.
